# Determination and Prediction of Time-Varying Parameters of Mooney–Rivlin Model of Rubber Material Used in Natural Rubber Bearing under Alternating of Aging and Seawater Erosion

**DOI:** 10.3390/ma16134696

**Published:** 2023-06-29

**Authors:** Wanbao Niu, Yanmin Li, Yuhong Ma, Guifeng Zhao

**Affiliations:** 1 Earthquake Engineering Research & Test Center, Guangzhou University, Guangzhou 510405, China; nwb0320@163.com; 2 Guangdong Key Laboratory of Earthquake Engineering & Applied Technique, Guangzhou 510405, China; zgfzth@gzhu.edu.cn; 3 Key Laboratory of Earthquake Resistance, Earthquake Mitigation and Structural Safety, Ministry of Education, Guangzhou 510006, China; 4 School of Civil Engineering, Guangzhou University, Guangzhou 510006, China

**Keywords:** effect of alternating aging and sea corrosion, natural rubber isolation bearings, rubber material, Mooney–Rivlin model parameters, nonlinear auto-regressive

## Abstract

In this paper, we examined the parameters of the Mooney–Rivlin model based on the effects of alternative aging and sea corrosion tests for natural rubber bearings and rubber materials in seawater. The model parameters for rubber material used in natural rubber bearings were determined using the least-squares method. Meanwhile, the time-varying law formula of the Mooney–Rivlin model parameters of rubber were fitted, and the fitting and calculated values were compared. Both fitting values and calculated values coincide with each other well. Then, the rubber material parameters were predicted based on the calculated results and combined with nonlinear auto-regressive (NAR). The predicted values were compared with both the fitting and calculated values. The average deviations between predicted and fitting values for *C*_10_ and *C*_01_ were 2.6% and 5.1%, respectively, and average deviations between predicted and calculated values for *C*_10_ and *C*_01_ were 5.2% and 4.1%. Compared results show that the predicted values are in good agreement with both the fitting and calculated values; meanwhile, the proposed time-varying law formula of the Mooney–Rivlin model parameters of rubber material have been well verified.

## 1. Introduction

Rubber isolation bearings are widely used in offshore bridge engineering to improve seismic safety [1]. However, the research on the life cycle design theory of offshore bridges has mainly concentrated on the durability of concrete piers. For example, Cheewaket et al. [2] studied the long-term chloride binding capacity of fly ash concrete under marine conditions. They found that the chloride binding capacity, in relation to the total chloride content, increased with the increased fly ash content in the concrete. Li [3] studied the durability of concrete bridge piers in a marine environment. Macia and Mirza [4] examined the sustainability and durability requirements of a conventional bridge structure subjected to various mechanical, natural, and man-made loads and a cold aggressive environment. Their study focused on the performance of various materials and structural components over the design service life of the bridge. In addition, the research on the durability of rubber bearings has mainly focused on aging and creep properties because studies have long shown that rubber has aging and creep problems [5]. However, few studies have examined the changing laws of the mechanical properties of rubber isolation bearings during their overall life cycle. Mott [6] studied the aging characteristics of rubber materials under various air and ocean temperatures. Gu and Itoh [7] conducted a series of environmentally accelerated aging tests to investigate the aging characteristics of natural rubber and high damping rubber at the material level. Their results showed that thermal oxidation was the most significant degradation factor and that after a significant period, the stress applied to the 100% strain can be 200% higher than the stress at time zero. Itoh et al. [8] performed thermal oxidation tests on natural rubber blocks at various temperatures using the modulus profiling method and established an appropriate aging model based on the data and phenomena of the tests, which were used to predict the aging characteristics of the natural rubber bearings. Kim et al. [9] studied the influence of thermal aging on the mechanical properties of laminated rubber bearings under accelerated exposure conditions of 70 °C for 168 h and quantitatively determined the property changes of rubber bearings aged mainly by heat. Finite element analysis is another important approach for studying the performance of structures and rubber bearings, because it can overcome the limitations of the laboratory condition and field measurement methods. The rubber constitutive model and accuracy of the material parameters used in the finite element model directly influence the precision of the simulation results. However, it is difficult to calculate the mechanical properties of rubber materials due to their complex molecular properties, such as the dual non-linear nature of rubber materials in relation to the geometric, temperature, load, time, and related factors [9,10,11,12]. Additionally, the Mooney–Rivlin model is suitable for examining moderate deformation and has been widely applied in finite element analyses of rubber bearings [13,14,15]. Hence, further finite element simulations are needed to understand how adverse weather conditions affect the parameters of the Mooney–Rivlin model for the rubber used in rubber isolation bearings.

Since the 19th century, numerous research projects have examined the constitutive properties of rubber. Boyce [16] noted that a number of theoretical constitutive models have been established for studying the properties of rubber. Rivlin [11] proposed that rubber is isotropic (i.e., the material properties are identical in all directions) and that the strain energy density function is converted into a series, which provided the theoretical basis for the development of the Mooney–Rivlin model. Ha-Anh and Vu-Khanh [17] conducted a neoprene hot-oxygen aging test to investigate the variations in the constants of the Mooney–Rivlin model over time and found that the model parameters were affected by the aging of the rubber material. 

Zhong et al. [18] conducted a uniaxial tensile test of rubber materials based on a combined Ex-ln (i.e., exponential-natural logarithmic) hyperelastic constitutive model and generalized viscoelastic method and proposed a hyperelastic constitutive model for describing the mechanical responses of rubber materials under different strain rates. Zuo and Xiao [19] used a theoretical derivation method to obtain the formulae of the material coefficients of a Mooney–Rivlin model with the same hardness in the range of small deformation. Li et al. [20] studied the effects of aging on the material constants of a Mooney–Rivlin constitutive model for rubber isolation bearings. Zhao et al. [21] conducted aging and marine corrosion tests on seismic isolation bearings to study the performance deterioration law of the bearings and developed a modified Mooney–Rivlin constitutive model for rubber materials. 

Aging is widely accepted to be the main factor leading to the deterioration of rubber isolation bearings and the Mooney–Rivlin model is the most commonly used approach for examining the behavior of the rubber materials used in rubber bearings. However, few studies have examined the parameters of the Mooney–Rivlin models of rubber isolation bearings. Moreover, the research on the life-cycle design theory for offshore isolated bridges has mostly focused on the durability of concrete bridge piers and the durability of the rubber bearings with respect to aging and creep. However, rubber bearings used in offshore or sea-crossing bridges are faced with complex and harsh marine environments. Rubber bearings in splash and tide zones will not only be affected by aging caused by ultraviolet light, temperature, and ozone in the air, but also by the marine erosion caused by sea water, wind and fog. Therefore, rubber bearings used in offshore or sea-crossing bridges easily suffer from the effect of alternating aging and sea corrosion during its service life. And the performance of rubber bearing will deteriorate or even fail due to the long-term effect of alternating aging and sea corrosion. However, few studies have examined the performance of seismic isolation bearings under a marine environment or the performance of isolation bearings under the effect alternating of aging and sea corrosion. Hence, the performance deterioration laws of rubber isolation bearings and the corresponding rubber materials under the effect alternating of aging, sea corrosion, and other marine environments remain largely unknown.

In this paper, we use experimental data on rubber materials under the alternating effect of aging and sea corrosion and the least-squares fitting method to obtain the time-dependent laws of the Mooney–Rivlin model parameters. Then, the Mooney–Rivlin model parameters are predicted by NAR. And the accuracy of the constants used in the Mooney–Rivlin model is then verified through comparing the predicted values with both the fitting and calculated values. The results provide a theoretical basis for further research on the performance of rubber isolation bearings under complex marine environments and the design, use, and maintenance of offshore isolated bridges.

## 2. The Mooney–Rivlin Model

The classic Mooney–Rivlin model for examining the constitutive properties of rubber is based on the following two assumptions [15,22,23]: (1) the rubber is incompressible and isotropic prior to deformation, and (2) the rubber follows Hooke’s law in simple shear. Hooke’s law is also observed when shearing is superimposed on a plane perpendicular to the uniaxial tension or compression axis. But (2) is not true for very large shear, where the normal strain matters. The Mooney–Rivlin model is suitable for simulating the mechanical properties of most rubber materials under small or medium deformation. The typical strain energy density function is shown in Equation (1) [15]:

(1) $$W=C_{10}(I_{1}-3)+C_{01}(I_{2}-3)+\frac{1}{D_{1}}{\left(J-1\right)}^{2}$$

where W represents the strain potential energy, $I_{1}$, $I_{2}$ are invariants of the deviatoric strain tensor, $C_{10}$, $C_{01}$ and $D_{1}$ are material constants, which can be determined from material tests of the rubber, and J is the elastic volume ratio.

## 3. Introduction for the Alternating Aging and Seawater Erosion Test

**Figure 1:**
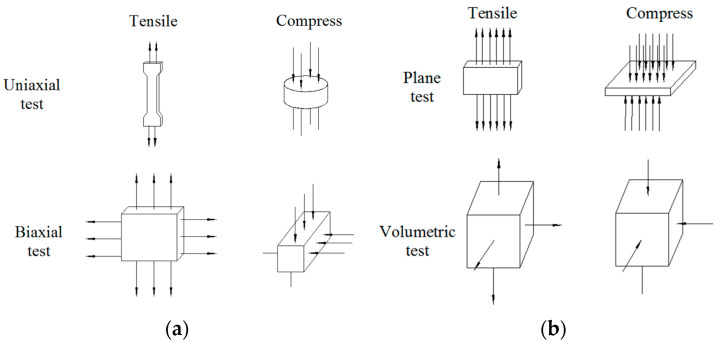
Eight common tests for rubber materials (**a**). Uniaxial test and biaxial test (**b**). Plane test and volumetric test.

The natural rubber bearings used in offshore isolated bridges face an extremely complex marine environment and can be affected by numerous factors. Furthermore, the weather conditions, such as sun, rain, sea breezes, sea waves, tides, and other factors, contribute to the effect of alternating aging and sea corrosion. To establish constitutive models of the rubber materials, the corresponding tests need to be conducted on the rubber materials. Eight kinds of tests are commonly used (see Figure 1): the uniaxial and biaxial tension and compression tests, and the plane stretching, plane compression, volumetric tensile, and compression tests [24,25].

**Figure 2:**
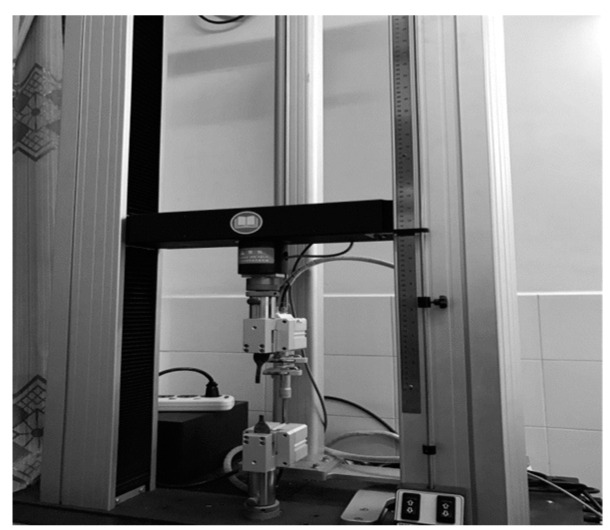
Tensile testing machine for rubber materials.

Except for the first type of test, the other seven test techniques are at the research stage and are very immature. Thus, the uniaxial tensile test has been widely used in China and other countries to measure the mechanical properties of rubber. In this paper, the uniaxial tensile test is also used to test the performance of the rubber material (see Figure 2). The tests mainly focus on the influence of the effect of alternating aging and sea corrosion on the rubber material parameters. Accelerated alternating aging and sea corrosion tests involving the continuous and repeated drying and soaking of rubber bearings and rubber sheets comprised of the same rubber material were conducted. The specimens were aged for twice as long as they are soaked in seawater to match the 2:1 ratio of sunny to rainy days observed in ten coastal cities in China [26].

### 3.1. Test Condition

**Figure 3:**
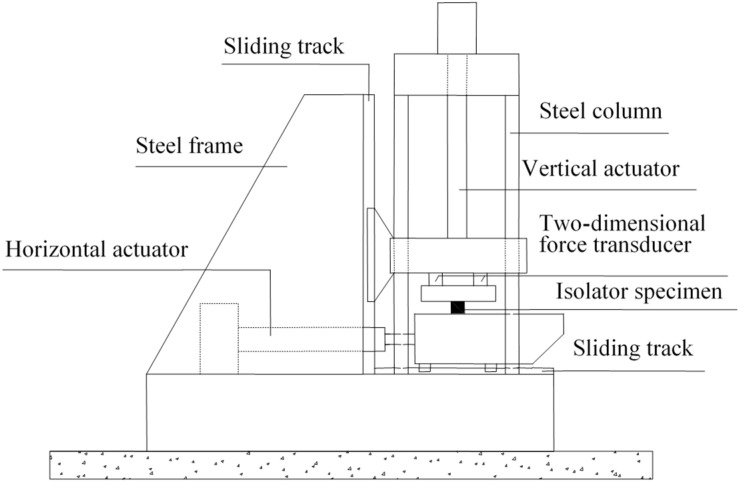
The tension-compression-shear set-up.

**Figure 4:**
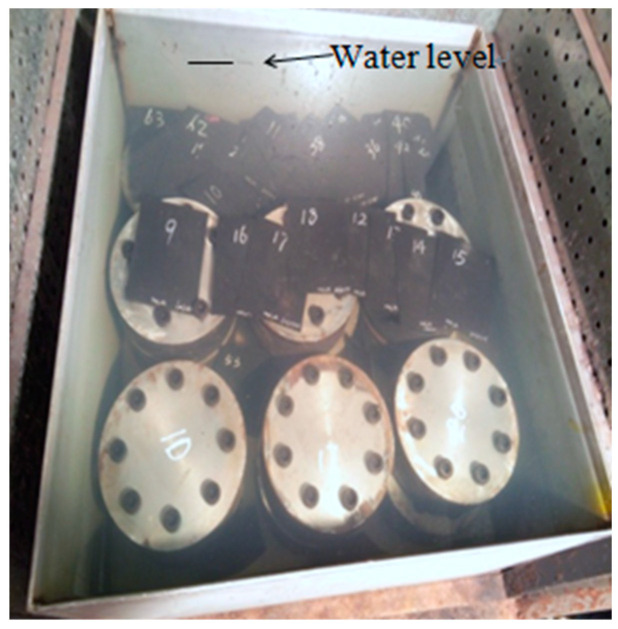
The specimens soaked in seawater.

**Figure 5:**
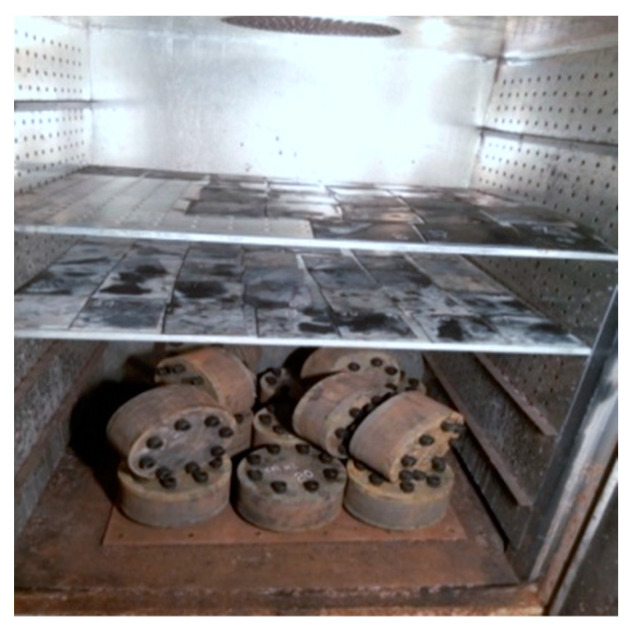
The specimens dried in the thermal aging box.

**Figure 6:**
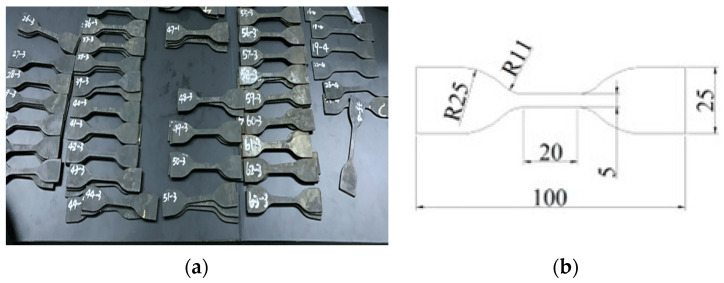
Dumbbell specimens of the rubber material (**a**). Dumbbell specimens; (**b**). dimensions of dumbbell specimens.

Before conducting the alternating aging and sea corrosion test, the performance of 20 natural rubber bearings and the mechanical properties of the rubber material were measured using the tension-compression-shear test set-up (see Figure 3) and a tensile testing machine (see Figure 2), respectively. The rubber material tensile testing procedure was according to ISO 37: 2005, IDT [27], the movement speed of the dripper was 500 mm/min, and the changes in test length and force were continuously monitored throughout the entire test process. Next, all of the specimens were soaked in an artificial seawater test box with a constant temperature of 80 °C for 1 day. The specimens were then dried in a thermal aging box with a constant temperature of 80 °C for 2 days (see Figure 4 and Figure 5). So, the overall testing period under the effect of alternating aging and sea corrosion comprised three days, and it took 60 days to complete all of the tests. During the effect of alternating aging and sea corrosion test, 63 rubber sheets (i.e., a total of 189 dumbbell specimens, see Figure 6) were removed from the test box every three days for testing and three rubber sheets were sampled each time. The hardness, tensile stress, elongation at break, and tensile strength of the samples were tested. The dimensions of the rubber sheets were 150 mm × 116 mm × 2 mm (length × width × thickness). The dumbbell specimens were cut from rubber sheets using an “I” type cutter [27].

The Arrhenius transformation equation [26] was used to convert the test time to the actual physical time:

(2) $$\ln\frac{t_{real}}{t_{}}=\frac{E_{a}}{R}(\frac{1}{T_{real}}-\frac{1}{T_{test}})$$

where $E_{a}$ represents the reaction activation energy (kJ/mol·K), R is the gas constant (8.3 [J/mol·K]), which is only dependent on the unit and is independent of the type of gas, $T_{real}$ is the absolute temperature in the actual marine environment (K), $T_{test}$ is the absolute temperature in the effect of alternating aging and sea corrosion test (K), $t_{real}$ is the actual service time for rubber isolation bearings in an actual marine environment, and t is the test time.

An artificial acceleration test approach was used, given the operability and limitations of the effect of the alternating aging and sea corrosion test. A real environmental temperature of 20 °C was simulated, which was obtained by calculating the average temperature of ten coastal cities in China, as well as an activation energy of 85 kJ/mol·K, and a test temperature of 80 °C. The results of Equation (2) indicated that the speedup ratio of this test was 376 (i.e., a test time of one day for the rubber bearing approximately simulated a service time of 376 days in an actual marine environment). In other words, a test time of one day for the specimens approximately simulated one year in a marine environment. Thus, the specimens were tested for 60 days to examine the performance change laws of the rubber bearings and rubber materials used in an offshore isolated bridge under an actual alternative aging and sea corrosion environment for 60 years. The parameters of the effect of alternating aging and sea corrosion test are shown in Table 1, and the test conditions for the rubber materials and rubber bearings are shown in Table 2.

### 3.2. Test Results for the Rubber Materials and Rubber Bearings

**Figure 7:**
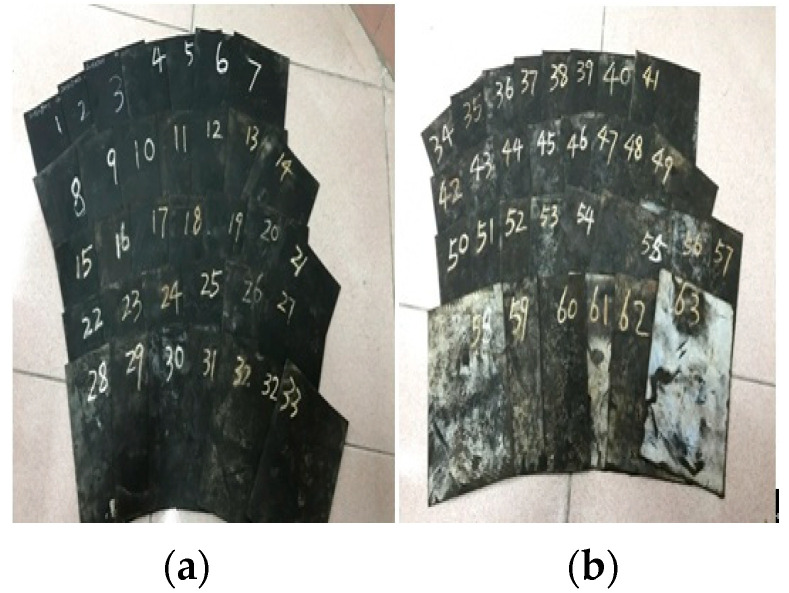
The rubber sheets after the alternative aging and sea corrosion test for (**a**) 0 to 30 days and (**b**) 31 to 60 days.

**Figure 8:**
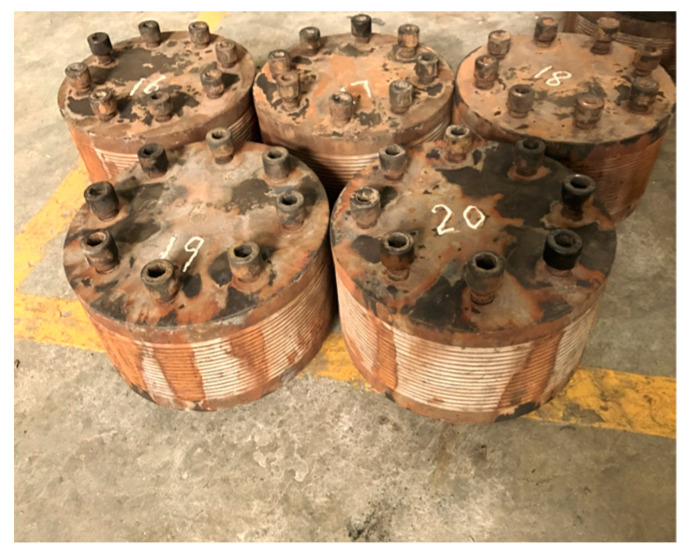
The rubber bearings after the 60-day alternative aging and sea corrosion test.

**Figure 9:**
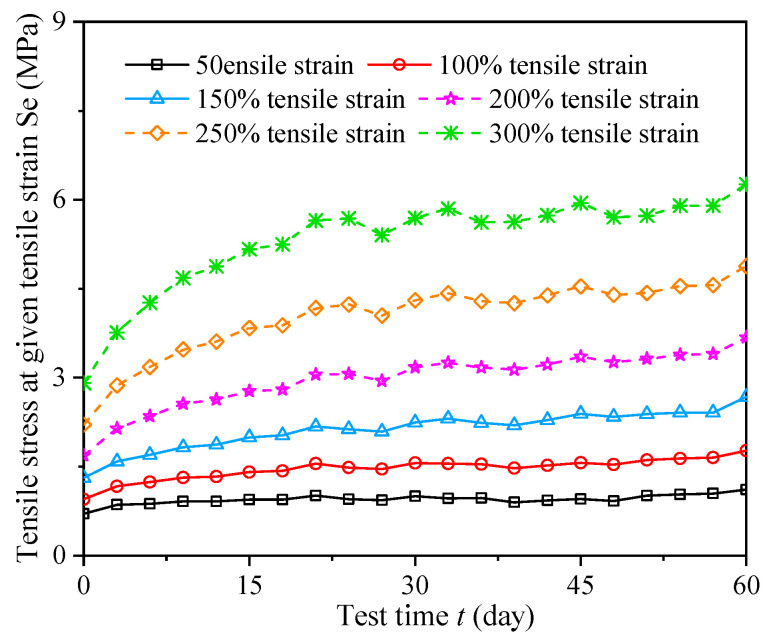
Effects of the alternating aging and sea corrosion on the tensile stress at different given tensile strains for the natural rubber material.

After completion of the 60-day tests, the appearance of the specimens changed. The surface of the rubber sheets was covered with salt (see Figure 7) and corrosion was found on the rubber bearing surface (see Figure 8). Because three rubber sheets (i.e., nine dumbbell specimens) were sampled every three days during the alternative aging and sea corrosion test, the test results for the mechanical properties of the rubber materials (see Figure 9) were averaged over nine dumbbell specimens. Figure 9 shows that the stress under different strains increases with time. The tensile stress at given tensile strains of 50%, 100%, 200%, and 300% increased by 58.20%, 86.39%, 118.02%, and 115.05%, respectively, after the 60-day tests. In the first 20 days of the alternative aging and sea corrosion test, the stress at a given elongation first increased quickly and then increased slowly, thus showing a periodic growth trend.

Although various properties of the 20 natural rubber bearings were tested, only the results for the horizontal and vertical stiffness of the bearings are shown in this paper (see Table 3). It can be seen from Table 3 that the mean values of the horizontal stiffness of the rubber bearings increased by 22.83% (considering the temperature correction), 23.18% (excluding the temperature correction), and 6.6%, respectively, after the 60-day alternative aging and sea corrosion test. The mean values were used as the test results for the stiffness of the rubber bearings in the following analyses.

## 4. Determination of the Rubber Materials Constants Using the Least-Squares Method

**Figure 10:**
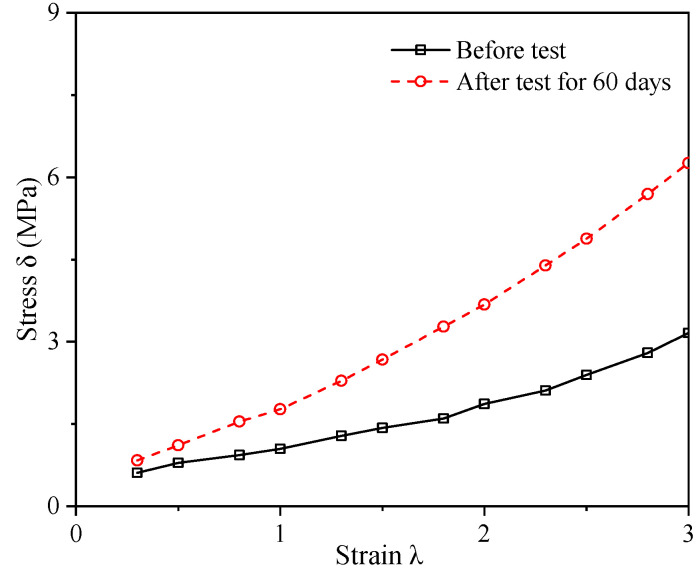
Tensile stress–strain relationships before and after the alternative aging and sea corrosion test.

The parameters of the rubber constitutive model are calculated using the least-squares method [20]. Experimental data from before and after the alternative aging and sea corrosion test are used to obtain the tensile stress–strain relationships (see Figure 10). The parameters for the rubber constitutive model are then calculated based on literatures [21,22,23,28]:

First, make $A_{1}=2(\lambda_{1}{}^{2}-1/\lambda_{1})$ and $B_{1}=\delta_{1}/A_{1}$, $E_{1}=1/\lambda_{1}$, so the relationships between the principal stresses, principal elongation ratios, and the deformation tensors invariant in Equation (3) in the literature [22] can be rewritten as Equation (3).

(3) $$\delta_{1}=\frac{2}{\lambda_{1}}(\lambda_{1}{}^{2}-\frac{1}{\lambda_{1}{}^{2}\lambda_{2}{}^{2}})(\frac{𝜕W}{𝜕I_{1}}+\lambda_{2}{}^{2}\frac{𝜕W}{𝜕I_{2}})=2(\lambda_{1}{}^{2}-\frac{1}{\lambda_{1}})(C_{10}+\frac{1}{\lambda_{1}}C_{01})$$

where $C_{10}=𝜕W/𝜕I_{1}$, $C_{01}=𝜕W/𝜕I_{2}$, $\delta_{1}$, $\delta_{2}$ and $\delta_{3}$ are the principal stresses, and $\lambda_{1}$, $\lambda_{2}$ and $\lambda_{3}$ are the principal elongation ratios.

(4) $$B_{1}=C_{10}+E_{1}\times C_{01}$$

Then, $A_{1}$, $B_{1}$, and $E_{1}$ can be obtained by the $\lambda_{i}$ and $\delta_{i}$ in Figure 10. And the calculations of $A_{1}$, $B_{1}$, and $E_{1}$ before and after the 60 days test are shown in Table 4 and Table 5.

**Figure 11:**
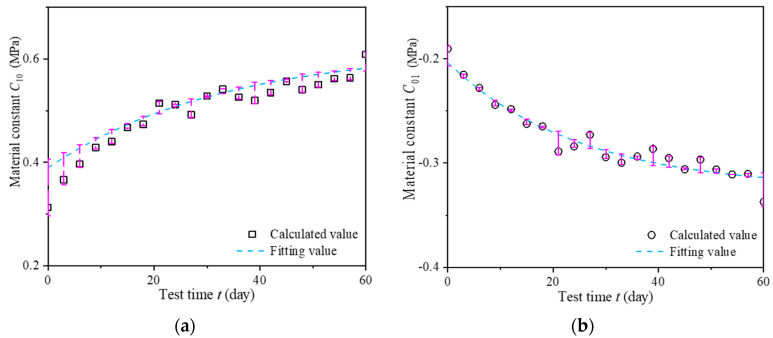
Effect of alternating of aging and seawater erosion on rubber material constants (**a**) *C*_10_ and (**b**) *C*_01_.

**Figure 12:**
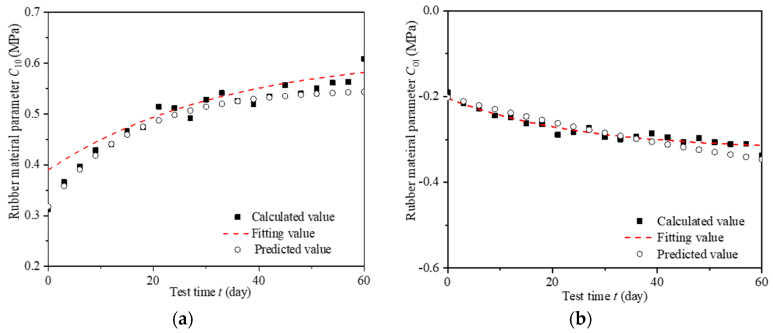
Prediction and verification of time-varying parameters of Mooney–Rivlin model for LNR rubber material. (**a**) *C*_10_ (**b**) *C*_01_.

Then, the rubber material constants *C*_10_ and *C*_01_ are then obtained, which are based on the calculations of $A_{1}$, $B_{1}$, and $E_{1}$ during the alternative aging and sea corrosion test. The change laws of material constants *C*_10_ and *C*_01_ with the alternative aging and sea corrosion times are given in Figure 11 and Figure 12 and Equations (5) and (6).

(5) $$C_{10}=0.62-0.23\times e^{-0.03\times t}$$

(6) $$C_{01}=-0.325+0.121\times e^{-\frac{t}{25.051}}$$

where *t* is the test time, which can be converted to the actual service time in an actual marine environment by Equation (2). The material constants *C*_10_ and *C*_01_ before and after the 60-day tests are calculated according to Equations (5) and (6).

Figure 11 shows that rubber material constants *C*_10_ and *C*_01_ increase linearly and exponentially, respectively, with time. The maximum error between the calculated results and fitted results for *C*_10_ is 29.75%. The majority of the error is within 10%, with a minimum error of 0.02% and an average error of 4.92%. The maximum error between the calculated results and fitted results for material constant *C*_01_ is 7.48%. The majority of the error is within 5%, with a minimum error of 0.13% and an average error of 2.41%. Thus, the fitted results are in good agreement with the calculated results. As previously mentioned, Equations (5) and (6) can be used to describe the change laws of the rubber material constants with time.

The material constant $D_{1}$ can be obtained by applying Equation (7):

(7) $$E_{\infty }=2/D_{1}$$

where $D_{1}$ is incompressible constant of rubber material.

The bulk elastic modulus *E*_∞_ can be determined based on [28,29] and Table 6.

## 5. Nonlinear Auto-Regressive (NAR) Neural Network Prediction and Verification of Mooney–Rivlin Model Parameters

The NAR neural network is a dynamic neural network model based on time series, and has feedback and memory functions [30,31]. Some studies have pointed out that the NAR model has the advantages of modeling and simulation of dynamic changes of time series, and its prediction effect is good in short time [32,33]. Therefore, the NAR neural network was used to establish the regression prediction model of the rubber material Mooney–Rivlin model parameters based on the time series of alternating aging and seawater erosion in this research. The NAR neural network algorithm is used to predict the Mooney–Rivlin model parameters *C*_10_ and *C*_01_ of rubber material for LNR at each test time point. Then, the predicted value of the NAR neural network (referred to as the predicted value) at each test time node was compared with its calculated and fitting value, as shown in Figure 12.

Figure 12 shows that the predicted values of Mooney–Rivlin model parameters *C*_10_ and *C*_01_ of rubber materials for LNR are in good agreement with their calculated values and fitting values. Comparison deviations are shown in Table 7. Thus, the accuracy and rationality of the time-varying law of rubber material Mooney–Rivlin model parameters obtained above are verified.

## 6. Conclusions

In this paper, we investigated the effects of alternating aging and sea corrosion on natural rubber material constants *C*_10_ and *C*_01_. The material constants *C*_10_ and *C*_01_ are determined by applying a least-square fit to the experimental data, and the relationship between the alternative aging and sea corrosion time and the material constants are derived. Furthermore, the NAR model was used to predict the rubber material parameters of Mooney–Rivlin for LNR. Then, the predicted results were compared with their calculated and fitting values to verify the accuracy of the proposed time-varying law of rubber material parameters of the Mooney–Rivlin model (see Formulas (8) and (9)). The primary conclusions are as follows.

(1) The effect of alternating aging and sea corrosion affect the Mooney–Rivlin model constants for rubber. The *C*_10_ for rubber increases exponentially with the alternating of aging and sea corrosion test time, whereas the *C*_01_ decreases nearly exponentially with the alternative aging and sea corrosion test time.
(2) The results of Mooney–Rivlin parameters predicted by the NAR model are in good agreement with their calculated and fitting results, which well verified the accuracy of the proposed time-varying formula of Mooney–Rivlin parameters under alternating of aging and seawater erosion.
(3) The Mooney–Rivlin model for natural rubber materials considering the effect of alternating aging and sea corrosion proposed in this paper provides a convenient approach for analyzing rubber bearings and other structures. The model can also provide a good theoretical method for studying the effect of alternating aging and sea corrosion on the performance of seismic isolation bearings and offshore isolated structures during their overall life cycle. The results obtained can further provide a theoretical basis for the seismic design, performance evaluation, and maintenance of isolated bridge structures under alternating aging and sea corrosion.

To sum up, the effect of alternating aging and seawater erosion has an important influence on the properties of rubber materials used in natural rubber bearings. And the Mooney–Rivlin model parameters were also impacted by this erosion factor. In this paper, the predicted values of the Mooney–Rivlin model parameters are in good agreement with both the fitting and calculated values; meanwhile, the proposed time-varying law formula of the Mooney–Rivlin model parameters of rubber materials were well verified.

## Figures and Tables

**Table 1:** Test parameters of the rubber bearings and rubber materials used in the alternating aging and sea corrosion test.

Speedup Ratio of the Alternative Aging and Sea Corrosion Test	Time Ratio of Drying and Soaking	Total Test Time (Days)	Equivalent to the Actual Service Time under the Alternating Aging and Sea Corrosion Environment (Days)	Test Temperature (°C)	Equivalent to the Actual Environment Temperature (°C)
376	2:1	60	60	80	20

Note: Ingredients of the artificial seawater used in the tests (g/kg): NaCl—23.5; MgCl_2_—5; Na_2_SO_4_—4; CaCl_2_—1.1.

**Table 2:** Test method and test conditions for the rubber bearings and rubber materials.

Test Specimens and Number	Test Content	Test Method	Sampling Method	Specimen
Rubber sheets: R1–R63	(1) Hardness; (2) Tensile stress at a given tensile-strain: 50%, 100%, 150%, 200%, 250%, 300%; (3) Elongation at break; (4) Tensile strength	(1) The specimens are soaked for 1 day in artificial seawater heated to 80 °C in an aging box; (2) Then, the specimens are aged for 2 days in the aging box at 80 °C without artificial seawater; (3) Test is conducted in the above cycle	Sampling three specimens every 3 days	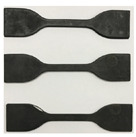

**Table 3:** Test results for the stiffness of the rubber bearings.

Project	*K*_v-test_ (kN/mm)	*K*_h-test_ (kN/mm)	*K*_h-temp_ (kN/mm)
Before test	380.4	0.3521	0.3559
	339.62	0.3041	0.3074
	321.4	0.3189	0.3224
	338.25	0.3104	0.3138
	364.63	0.3455	0.3493
After the 60 day alternative aging and seawater erosion	-	0.3837	0.3868
	381.46	0.4155	0.4189
	365.55	0.3884	0.3916
	368.6	0.3883	0.3915
	-	0.4332	0.4367

**Table 4:** $A_{1}$, $B_{1}$, and $E_{1}$ before test (i.e., test for 0 days).

Strain *λ*	*A*_1_	*B*_1_	*E*_1_	Stress *δ* (MPa)
0.3	−6.4867	−0.0931	3.3333	0.604
0.5	−3.5000	−0.2254	2.0000	0.789
0.8	−1.2200	−0.7648	1.2500	0.933
1	0.0000	-	1.0000	1.042
1.3	1.8415	0.6978	0.7692	1.285
1.5	3.1667	0.4503	0.6667	1.426
1.8	5.3689	0.2978	0.5556	1.599
2	7.0000	0.2660	0.5000	1.862
2.3	9.7104	0.2173	0.4348	2.11
2.5	11.7000	0.2048	0.4000	2.396
2.8	14.9657	0.1869	0.3571	2.797
3	17.3333	0.1821	0.3333	3.157

**Table 5:** $A_{1}$, $B_{1}$, and $E_{1}$ after testing for 60 days.

Strain *λ*	*A*_1_	*B*_1_	*E*_1_	Stress *δ* (MPa)
0.3	−6.48667	−0.12882	3.333333	0.836
0.5	−3.5	−0.31682	2	1.109
0.8	−1.22	−1.26708	1.25	1.546
1	0	-	1	1.767
1.3	1.841538	1.242612	0.769231	2.288
1.5	3.166667	0.845122	0.666667	2.676
1.8	5.368889	0.609599	0.555556	3.273
2	7	0.525397	0.5	3.678
2.3	9.710435	0.452086	0.434783	4.390
2.5	11.7	0.41688	0.4	4.877
2.8	14.96571	0.380618	0.357143	5.696
3	17.33333	0.361062	0.333333	6.258

**Table 6:** Compressibility coefficient for the alternative aging and sea corrosion test.

Alternative Aging and Sea Corrosion Test Time (Days)	Test Value of Hardness	*E*_∞_ (MPa)	*D*_1_
0	41	1003	0.001994
3	43	1009	0.001982
6	43	1009	0.001982
9	43	1009	0.001982
12	44	1012	0.001976
15	44	1012	0.001976
18	45	1015	0.00197
21	46	1018	0.001965
24	46	1018	0.001965
27	45	1015	0.00197
30	46	1018	0.001965
33	47	1021	0.001959
36	47	1021	0.001959
39	47	1021	0.001959
42	47	1021	0.001959
45	47	1021	0.001959
48	48	1024	0.001953
51	48	1024	0.001953
54	48	1024	0.001953
57	48	1024	0.001953
60	50	1030	0.001942

**Table 7:** Deviations between predicted, calculated and fitting values.

Rubber Material Parameter	Deviations between the Predicted and Calculated Values (%)		Deviation between the Predicted and Fitting Values (%)	
	Maximum Deviation	Average Deviation	Maximum Deviation	Average Deviation
*C*_10_	10.6	2.6	18.5	5.2
*C*_01_	9.8	5.1	10.3	4.1

## Data Availability

Not applicable.
